# Influence of Post System and Geometry on Radiant Energy Transmission Through the Side of Different Prefabricated Fiber Post Systems

**DOI:** 10.3390/polym18121429

**Published:** 2026-06-08

**Authors:** Abdulaziz M. Alqarni, Ahmad Y. Imam, Thamer Y. Marghalani

**Affiliations:** 1 Department of Prosthodontics, Dental Specialist Center, King Fahad General Hospital, Ministry of Health, Jeddah 22251, Saudi Arabia; 2 Department of Oral and Maxillofacial Prosthodontics, Faculty of Dentistry, King Abdulaziz University, Jeddah 21589, Saudi Arabia; ahmad.y.imam@gmail.com (A.Y.I.); tmarghalani@kau.edu.sa (T.Y.M.)

**Keywords:** fiber post systems, transmitted light radiant energy, resin cement polymerization, light transmission

## Abstract

Adequate polymerization of resin cements in fiber post restorations depends on effective light transmission, which is influenced by post type, design, and length; however, variations in transmitted light radiant energy (TLRE) among systems may compromise bonding and long-term outcomes, and comparative evidence remains limited. This study evaluated radiant exposure and TLRE transmitted by different fiber post systems across varying post lengths. Four fiber post systems (Easy Post, RelyX Fiber Post, iLumi Super Fiber Post, and EZ-Fit Translucent Post) and a glass rod as a control group were tested (*n* = 40). TLRE was measured through standardized side openings at 1 mm increments after 40 s of light curing and recorded with a radiometer, while the post-microstructure was analyzed using scanning electron microscopy. Hierarchical multiple linear regression was used to assess the effects of post system and length on TLRE. TLRE differed significantly among systems (*p* < 0.001), with post type and length explaining a substantial proportion of variance; length was a significant negative predictor across all systems. Only 1.20–7.19% of coronal TLRE reached post surfaces. The iLumi post maintained TLRE > 200 mJ/cm^2^ along its length, and microstructural differences were observed between smooth and serrated designs. Fiber post type, configuration, and length significantly influence TLRE, which decreases with increasing length, highlighting the importance of appropriate post selection to optimize resin cement polymerization and clinical performance.

## 1. Introduction

Intra-radicular post systems are frequently required to rehabilitate endodontically treated teeth, providing support for overlying fixed dental prostheses [1]. Among these, prefabricated fiber posts—such as glass or composite-reinforced designs—have gained widespread adoption due to their esthetic appearance, similarity in modulus of elasticity to dentin and resin cement [2,3,4,5,6], favorable stress distribution [7,8,9], and effective light transmission [10]. Prefabricated posts are classified according to their type, shape, and surface configuration [2,11,12], and commonly include methacrylate or epoxy, polyethylene, quartz/glass, and unidirectional carbon fiber-reinforced posts [13]. Encasing fibers within an epoxy or methacrylate matrix enhances their flexural and tensile strength [13,14,15].

The shape, surface configuration, and fiber orientation of posts significantly influence their mechanical and optical properties [16]. Common designs include tapered or parallel posts with smooth, serrated, or threaded surfaces [2], and fibers may be oriented unidirectionally or multidirectionally. Higher fiber concentration within the matrix correlates with increased tensile strength and improved light transmission [16,17,18].

Despite these advancements, effective bonding and retention of fiber posts remain challenging, with debonding from the root canal wall being the most frequent failure mode [19,20]. Retention is influenced by the depth of cement polymerization, and prefabricated posts are commonly cemented using auto- or dual-polymerizing resin cements [21,22]. A direct correlation exists between the polymerization of resin-luting cement and its bond strength to dentin [23]. Complete polymerization requires effective light transmission from the photo-polymerizing unit to the deepest radicular regions [24], which is affected by the material composition and optical properties of the post [25]. Resin-based cements are selected for their compatible modulus of elasticity, favorable stress distribution, and optimal retention [26]. Dual-cured cements are particularly advantageous due to their extended working time and high degree of conversion even in the absence of sufficient light [27], although inadequate light exposure can compromise polymerization.

Light transmission through fiber posts varies in irradiance and radiant exposure [28,29,30]. Irradiance represents the radiant flux per unit area (mW/cm^2^) [25], while radiant exposure (or radiant fluence) is the total energy received per unit area over time (mJ/cm^2^) [31]. The influence of light curing on the degree of conversion (DC) of resin cement has been documented [28,29,32,33,34]; however, few studies have examined apical light intensity [28,35,36,37,38] or transmitted light radiant exposure (TLRE) through posts of different systems, lengths, and diameters [38,39].

Studies indicate that light transmission through translucent fiber posts is often insufficient for effective polymerization in deeper canal regions [28,35,36,37,38,39]. Significant reductions in luminous energy have been reported with increasing post depth [28], while higher translucency has been shown to enhance light transmission and improve resin cement polymerization [35]. Additionally, it has been observed that less than 40% of incident light passes through certain prefabricated posts even at a depth of 10 mm [36]. Similar research has shown that all tested post systems exhibit decreasing light intensity with increasing depth, with fiber-reinforced composite posts transmitting more light than high-translucent zirconium oxide or ceramic zirconia posts [37]. One investigation found that post diameter and length significantly predict TLRE, with larger diameters and shorter lengths allowing greater light transmission, whereas the specific post system had minimal impact [38]. Another study reported that radiant exposure along the sides of posts varied linearly but exhibited distinct peaks at 9–13 mm from the coronal end, with side light transmission differing from cross-sectional light transmission [39].

Overall, the degree of conversion and physical properties of resin cements are directly linked to radiant exposure during polymerization [40,41]. While this relationship is not strictly linear, DC stabilizes at energies exceeding 10 J/cm^2^ [42,43]. Excessive radiant energy may increase polymerization stress without significantly enhancing conversion [40,44,45]. These findings underscore the critical importance of understanding the light transmission characteristics of fiber posts to ensure effective cement polymerization, optimal bonding, and long-term clinical success.

Resin cements require light for polymerization, but access limitations in root canals can cause incomplete curing. Light transmission through fiber posts varies with system, configuration, and length, affecting cement effectiveness. Few studies have examined TLRE along post sides [38,39], leaving a gap in understanding how post types and designs influence curing efficiency. Therefore, the aim of this study was to determine the amount of radiant exposure (TLRE) emitted from the lateral aspect of different prefabricated post systems at various lengths.

### 1.1. The Null Hypothesis (H0)

There are no statistically significant differences in radiant exposure (TLRE) emitted by the different post systems across the various length levels.

### 1.2. The Alternative Hypothesis (H1)

Radiant exposure (or TLRE) emitted by different post systems shows statistically significant differences across various length levels.

## 2. Materials and Methods

**Figure 1:**
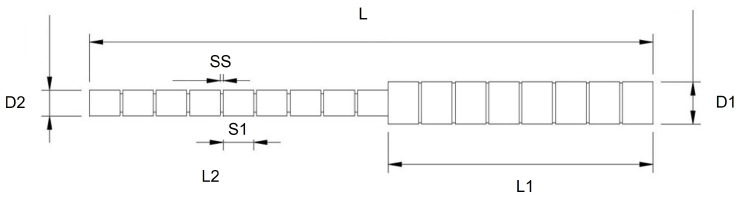
Characteristics of fiber post systems investigated.

The study evaluated 4 prefabricated fiber post systems, categorized by design as shown in Table 1 and Figure 1. A total of 40 specimens were included, with 10 specimens assigned to each post system.

### 2.1. Mold and Specimen Preparation

**Figure 2:**
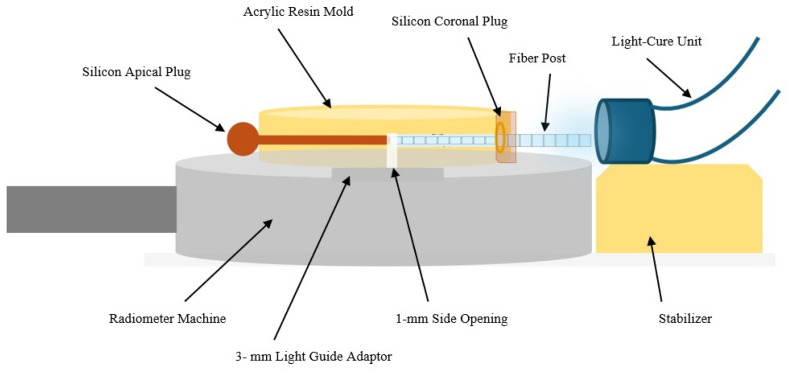
Schemic illustration of TLRE measurement process.

A custom mold was fabricated from temporary acrylic resin in shade A2 (c&b II temporary resin; Ivoclar Vivadent AG, Amherst, NY, USA) using an impression of a 3 mm light-guide adaptor (Figure 2). The mold featured a 1 mm side opening for TLRE measurement of the embedded fiber post. The mold was placed over a radiometer (ACCU-CAL 50-LED; Dymax Corp., Torrington, CT, USA), with the post held by a silicone stopper in a 2 mm hole diameter to allow sliding. The radiometer utilized an 8 mm light-guide setting to capture the maximum light intensity, or curing light irradiance, directly from the light-emitting diode (LED) curing unit (D-2000, APOZA Enterprise Co., Ltd., New Taipei, Taiwan). The irradiance recorded from the light probe was 637 mW/cm^2^, which corresponded to a total radiant exposure of 30.88 J/cm^2^ over 40 s. The wavelength spectrum emitted by the LED curing unit ranged from 430 to 490 nm. To guarantee that the intensity of the curing light remained consistently above 600 mW/cm^2^ throughout the testing procedures, the intensity was measured three times directly from the light probe tip before each post measurement.

### 2.2. TLRE Measurement

The examined post systems generally exhibited a two-thirds coronal parallel segment and a one-third apical tapered segment, whereas the EZ-Fit system demonstrated an inverse configuration. The parallel regions corresponded to a rectangular profile, and the tapered regions to an isosceles trapezoid. Serrations were identified in the iLumi system (2.3 mm × 4 with 0.5 mm steps and 1.7 mm × 3) and EZ-Fit system (1 mm × 16 with 0.1 mm step length).

The variation in diameter and configuration through the mold side hole was considered when calculating TLRE from the detector surface before entering the values into the regression analysis. The rectangle area was computed as follows:

Area of the rectangle = Width × Length

The area of the tapered sections was determined using the formula for the area of an isosceles trapezoid.

$$Area\;of\;isosceles\;trapezoid=\frac{1}{2}C\left(A+B\right)$$

**Figure 3:**
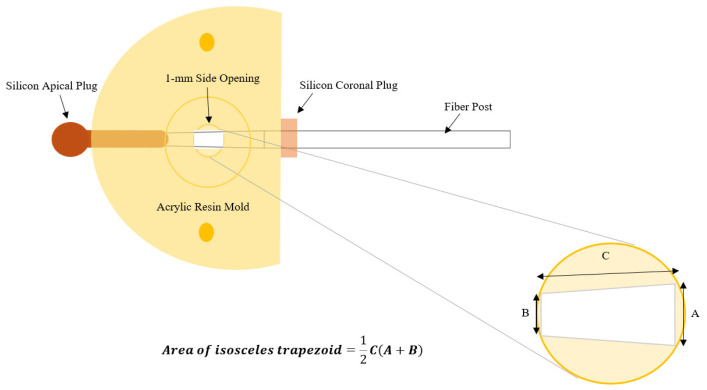
Schematic diagram illustrating the area measurement of an isosceles trapezoid in the taper section of a post through a side opening. A: post diameter, B: diameter of post at certain length, C: diameter of the circle.

The length of the post and its tapering portion as it passes through the mold determine the trapezoid’s long base, denoted by A in Figure 3, and its short base, denoted by B. The length and taper of the post may also be used to determine the base in Figure 3, and C represents the trapezoid height, which is about equal to the side hole diameter.

**Figure 4:**
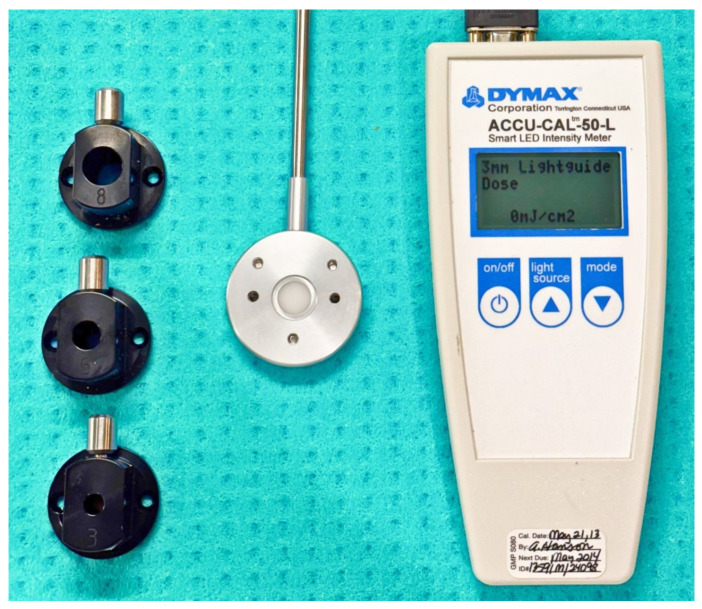
LED light radiometer with flood lamp sizes of 3, 5 and 8.

The LED radiometer features a changeable light-guide adaptor with an 8 mm sensor and adjustable settings to accommodate 3, 5, or 8 mm measurement areas (Figure 4). It operates in three modes: live light intensity (mW/cm^2^), peak light intensity (mW/cm^2^) proportional to the exposed area, and light energy (mJ/cm^2^), calculated as intensity multiplied by exposure time relative to the selected diameter setting.

The closest preset region that matches the side hole is selected as the area setting. Following that, the radiant energy was measured using this formula:

Radiant Exposure (actual area) = Radiant Exposure (area setting) × Area factor (actual area/predetermined area)

With the 8 mm light-guide adaptor, light output from the curing tip was measured, and radiant exposure was adjusted to the corresponding area to determine the energy reaching the coronal end of the post. This allowed calculation of the area factor, defined as the post end diameter divided by the 8 mm setting area. The proportions of TLRE emitted from the coronal end relative to the sides are reported in Table 2.

**Figure 5:**
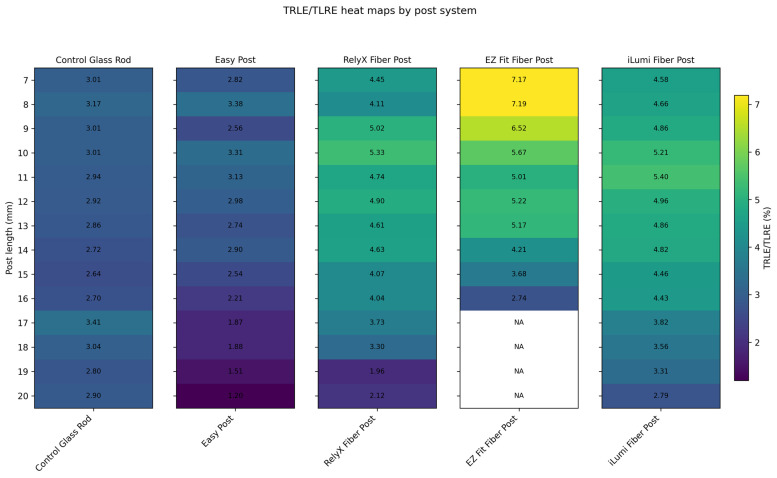
Heat map showing the TRLE Map (Mean Value) of each post system across the lengths.

The post was inserted into a mold with its apical end visible through the side opening. A silicone plug was used to simulate a gutta-percha at the apical end, and the stopper was relined with silicon (3M™ Express™ XT VPS Impression Material, 3M ESPE Dental Products, St. Paul, MN, USA) to prevent light leakage coronally. The LED curing tip was activated for 40 s, and side irradiance was measured using the radiometer. The TLRE was determined by multiplying the actual measured area by the closest preset holder area setting in the radiometer. The actual measured area was based on either the side opening or the diameter at the coronal end of the post. The ratio of the actual post diameter to the 8 mm setting area is used to calculate the peak intensity and dose at the coronal end for each type and size of prefabricated fiber post system used (Table 2 and Figure 5).

### 2.3. Micro-Structure Analysis

Longitudinal sections of all post systems were prepared using a low-speed rotary saw (IsoMet low-speed saw; Buehler; Lake Bluff, IL, USA) and cleaned with ethanol. Samples were mounted on scanning electron microscopy (SEM) with gold-conducting tape and coated with a thin gold layer using a vacuum sputter coater (Desk-I; Denton Vacuum Inc., Moorestown, NJ, USA). Coronal, middle, and apical sections were examined under SEM (Quanta FEG 650, FEI, Hillsboro, OR, USA) in secondary electron mode to analyze microstructural features.

### 2.4. Statistical Analysis

Preliminary statistical evaluations using the Shapiro–Wilk and Kolmogorov–Smirnov tests for normality, along with Levene’s test for homogeneity of variance, revealed no violations of the underlying assumptions. Descriptive statistics were generated for all variables. A three-step hierarchical multiple regression analysis was then conducted at a significance level of α = 0.05 to assess the influence of post system and post length on TLRE. In Step 1, the post system was entered into the baseline model as the initial predictor. In Step 2, the actual post diameter was added to evaluate its additional contribution. In Step 3, post length was introduced as a further predictor to determine its incremental effect. One-way ANOVA with Bonferroni post hoc analysis was used to identify specific group differences. All statistical tests were performed using SPSS (Version 28.0, IBM Corp., Armonk, NY, USA).

## 3. Result

**Figure 6:**
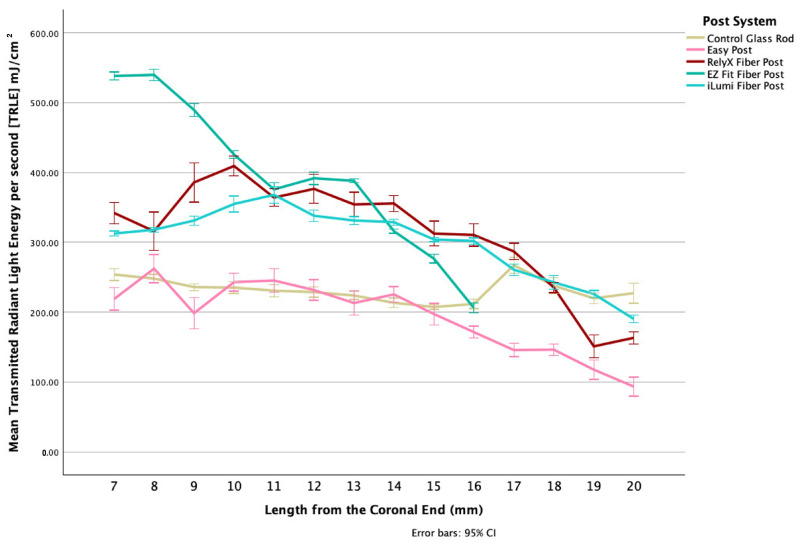
Mean TLRE mJ/cm^2^ by post system and length from coronal end.

Descriptive data showed that smooth posts (*n* = 420) had a mean advancement length of 13.50 (SD = 4.036) and TLRE of 245.54 mJ/cm^2^ (SD = 74.92), while serrated posts (*n* = 240) had 12.67 (SD = 3.735) and 339.83 mJ/cm^2^ (SD = 90.65); the Control Glass Rod recorded 13.50 (SD = 4.04) and 231.46 mJ/cm^2^ (SD = 19.74), reflecting substantial variability across parameters, detailed in Table 2 and Figure 6.

A post hoc power analysis was performed for the hierarchical multiple regression, and it showed an observed power of 1000 for the corrected model, intercepts, and each main effect and interaction. Regression analyses for smooth and serrated post configurations were conducted separately, and each was statistically significant (Table 3). For the analysis of smooth post systems, the predictor post system was entered hierarchically using the following sequence: Control Glass Rod, Easy Fiber Post, and RelyX Fiber Post. The hierarchical regression revealed that the model containing only the post system explained 19.1% of the variance in TLRE (R^2^ = 0.191, *p* < 0.001). The subsequent addition of Length from the Coronal End (mm) resulted in a significant model improvement, F(1, 417) = 168.77, *p* < 0.001, accounting for an additional 23.3% of the variance (∆R^2^ = 0.233). The final two-predictor model explained 42.4% of the total variance. A parallel analysis was performed for serrated post systems, entered in the sequence of Control Glass Rod, EZ-Fit Fiber Post, and iLumi Fiber Post. The initial model with the post system alone explained 22.8% of the variance (R^2^ = 0.228, *p* < 0.001). Adding Length from the Coronal End again provided a significant improvement, F(1, 377) = 216.96, *p* < 0.001, ∆R^2^ = 0.282. The final model for serrated posts accounted for 51.0% of the variance in TLRE.

The Length from the Coronal End (mm) significantly predicted side TLRE for all post systems (*p* < 0.05), as detailed in Table 2. For the Control Glass Rod, Length from the Coronal End was a significant predictor of TLRE (B = −1.286, t(138) = −3.21, *p* = 0.002), indicating that each additional millimeter from the coronal end reduced TLRE by 1.286 mJ/cm^2^. In the Easy Post system, Length from the Coronal End also significantly predicted TLRE (B = −10.625, t(138) = −15.93, *p* < 0.001), corresponding to a 10.625 mJ/cm^2^ decrease per millimeter. The RelyX Fiber Post showed a similar pattern, with Length from the Coronal End significantly predicting TLRE (B = −14.967, t(138) = −13.73, *p* < 0.001), resulting in a 14.967 mJ/cm^2^ reduction per millimeter. For the EZ-Fit Fiber Post, Length from the Coronal End strongly predicted TLRE (B = −35.150, t(98) = −38.32, *p* < 0.001), with each additional millimeter lowering TLRE by 35.150 mJ/cm^2^. Finally, the iLumi Fiber Post demonstrated a significant prediction of TLRE by Length from the Coronal End (B = −9.873, t(138) = −14.89, *p* < 0.001), reflecting a decrease of 9.873 mJ/cm^2^ per millimeter.

Overall, measurements showed that the TLRE transmitted and emitted from the post sides ranged between 93.37 and 538.28 mJ/cm^2^, indicating that only 1.20% to 7.19% of the original TLRE at the coronal end reached the post surface (Table 2).

### SEM Analysis

**Figure 7:**
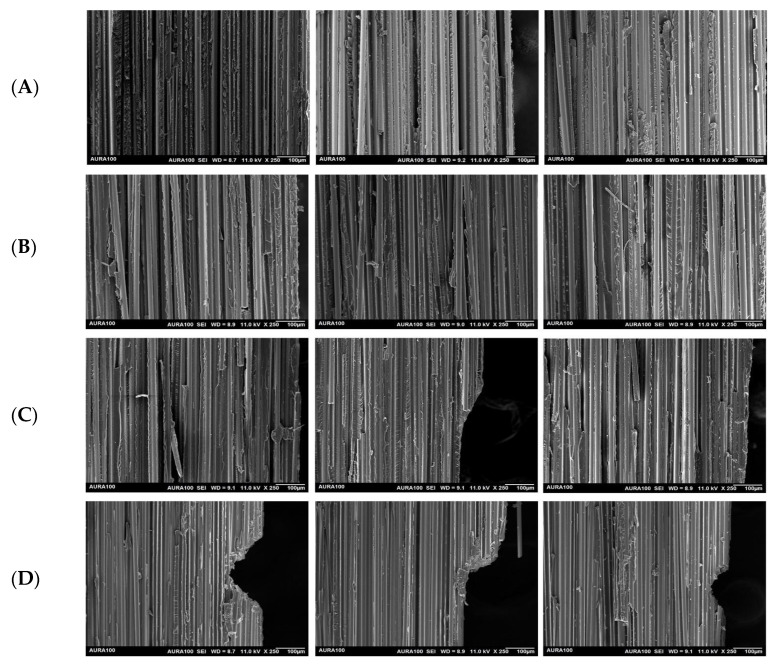
Scanning electron microscope images of tested post systems (left to right) at coronal, middle, and apical region. (**A**) Easy Post system. (**B**) Relyx post system. (**C**) iLumi post system. (**D**) EZ-Fit post system (magnification of ×100).

SEM analysis suggested structural differences between post systems. The smooth Easy Post appeared to exhibit variable fiber dimensions with relatively low resin content, while RelyX appeared to show more uniform fiber dimensions. Serrated posts featured fibers terminating at each serration step without clear continuation. EZ-Fit additionally appeared to exhibit densely packed fibers with comparatively low resin content (Figure 7).

## 4. Discussion

The null hypothesis—that TLRE per second is equal across all post systems at different advancement lengths—was rejected (*p* < 0.001), with differences attributed to variations in material composition, structural design, diameter, length, translucency, and surface texture, which influenced light scattering and absorption.

Hierarchical regression showed that the post system explained 19.1% of TLRE variance for smooth posts and 22.8% for serrated posts, while the addition of advancement length increased the explained variance to 42.4% and 51.0%, respectively, indicating that length strongly influences light transmission.

All post systems exhibited significant light attenuation with increasing depth, consistent with the Lambert–Beer Law [24] and previous studies [28,46,47]. EZ-Fit demonstrated the highest TLRE at 6–7 mm but declined sharply after 8 mm, falling to 200–300 mJ/cm^2^ radiant exposure at 16 mm.

RelyX peaked at 10 mm, then declined gradually and fell below 200 mJ/cm^2^ at 18 mm. iLumi peaked at 11 mm and maintained radiant exposure above 200 mJ/cm^2^ throughout the entire length. Easy Post had lower TLRE, dropping below 200 mJ/cm^2^ at 15 mm. The Control Glass Rod exhibited stable TLRE (200–300 mJ/cm^2^), indicating low attenuation. While 100 mW/cm^2^ is the minimum irradiance for curing thin cement layers under ceramic restorations [48], the observed radiant exposure values of 200–300 mJ/cm^2^ (equivalent to 5–7.5 mW/cm^2^ over 40 s) may enhance the polymerization and bonding of the fiber post; however, further research is needed.

Variations in post systems or brands significantly affect light transmission through fiber posts [35,37,39,49], with some limiting transmission and reducing cement polymerization [28,35,37,49]. Material composition and design also influence polymerization efficiency. Most glass fiber-reinforced posts consist of 40–65% fibers in dimethacrylate- or epoxy-based matrices [5,10,50,51,52]. Additives such as white or opaque materials may also affect light transmission [34], a factor that was not measured in this study. Furthermore, fiber orientation, diameter, matrix, and matching refractive indices (RIs) between fibers and resin enhance light conduction [18], with fibers acting as optical waveguides [53].

Prefabricated fiber posts usually contain many fibers embedded within a resin matrix. Fibers represent the largest proportion of fiber-reinforced posts, making up 40–65% of the volume [52]. Glass fibers are the most commonly used in fiber-reinforced posts due to their favorable mechanical properties and translucent appearance [5]. Increasing the number of fibers in the post improves light transmission. The differences in fiber orientation, diameter, matrix, and filler result in varying refractive indices, which affect light transmission along the post [10]. The resin matrix also helps to maintain fiber integrity and facilitate light transmission. However, when the matrix refractive index nearly matches the fibers, more light may pass through the post’s side [18]. The refractive index (RI) quantifies how much light bends as it travels through a material. In fiber posts, variations in RI between the resin matrix and reinforcing fibers significantly impact light transmission efficiency. The refractive index of the bulk resin matrix is influenced by the amorphous polymer’s refractive index, which can be estimated using the molar properties of a single polymer unit through techniques such as molar refraction. Another key optical property is the extinction coefficient, which describes how light attenuates as it propagates through the material. Both the refractive index and extinction coefficient are wavelength-dependent and influence absorbance, transmittance, and reflectance [18]. Fibers can serve as optical waveguides, conducting light throughout their length [53]. Optical fibers are made up of a central core surrounded by a protective outer cladding layer. Light can travel efficiently due to a waveguide effect when the core has a slightly higher refractive index than the cladding. The behavior of light in optical fibers is explained by wave theory, with mathematical calculations conducted over short distances to understand the distribution and intensity of the light throughout the fiber [53]. Different modes of light propagation exist in optical fibers, including guided, leaky, and cladding modes.

The guided mode minimizes light loss, while the leaky mode allows some light to escape through the cladding. Cladding mode, on the other hand, involves the light extending into the outer area, resulting in greater light degradation [53]. The number of guided modes available is influenced by the fiber design and the wavelength used, with shorter wavelengths allowing for more modes and higher light loss. For applications demanding efficient light transmission, the guided mode is recommended. Conversely, fibers designed for cladding mode are employed when light diffusion through the cladding is advantageous [53]. In dental applications, fiber posts are intended to enhance light transmission from their sides to effectively initiate and cure resin cements, making cladding mode optical fibers a suitable option for maximizing light exposure during polymerization. However, in the current study, all post systems were composed of glass fiber embedded in an epoxy matrix with varying percentages. These optical properties of the fiber post resin matrix were not measured.

Post diameter strongly affects light transmission in fiber posts [24,28,38,39]. All tested posts were approximately tapered from 1.35 mm coronally to 0.8 mm apically, and this tapering was considered in TLRE calculations. Marghalani [38] observed higher TLRE in posts of length shorter than 9–10 mm, with wider coronal and middle regions transmitting more light than the narrower apical area due to density and light scattering. Alkhallagi [39] tested a similar post system and found that each 1 mm increase in diameter reduced TLRE by 44.96 units, emphasizing the role of post geometry. However, differences in study design may explain TLRE variations. In the current study, 1 mm side openings limit the measurements to the sensor-facing surface, restricting the assessment of full lateral light transmission.

This study compared smooth and serrated post systems. Previous studies have suggested that serrations may reduce light transmission [28,35,36], with results varying by the post system and methodology used. In the current study, the tested serrated posts system (iLumi Super and EZ-Fit) had different serration designs. The EZ-Fit post system had the highest average TLRE but a sharp decline at 8 mm. However, all post systems showed a pattern of an initial increase, a mid-length peak, and an apical decline in TLRE. One study reported that shorter posts (<9–10 mm) have higher TLRE due to the larger diameters of the coronal and middle thirds [38]. The present study demonstrated that smaller serration steps may increase light transmission. The fiber orientation was discontinued at the serration step in both the EZ-Fit and iLumi post systems, allowing more light to pass through and scatter at the coronal end compared to the middle and apical thirds. The discontinuity in fiber alignment creates irregular surfaces that scatter light in multiple directions. This could enhance the light dispersion at the coronal region, leading to higher localized light intensity. In fiber posts, continuous fiber alignment helps guide the light efficiently along the length of the post. When the fibers are disrupted, light transmission may become less efficient, causing more attenuation as light moves toward the middle and apical thirds. In other words, while the first serration step may cause light to scatter, some of the transmitted light can still reach the next serration step through internal reflection, refraction within the resin matrix, and redirection by the remaining fiber structure. However, light intensity will likely decrease as it progresses deeper into the post. This may explain why the EZ-Fit post system had a high initial TLRE coronally and a subsequent sharp drop apically. In addition to serration-related fiber discontinuity, other factors such as fiber content, matrix composition, taper geometry, and manufacturer-specific optical properties may also contribute to the observed attenuation in TLRE.

TLRE along post sides depends on light intensity and exposure time, influencing DC, bonding, and cement strength. Marghalani [38] found a linear relation between TLRE and post dimensions, with higher TLRE in shorter posts (peak at 5 mm). Alkhallagi [39] reported peaks at 9–10 mm for FiberKleer and size 3 Postec Plus, and 13 mm for the smaller Postec Plus post system. In this study, Easy Post peaked at 8 and 11 mm, RelyX at 10 mm, iLumi at 11 mm, and EZ-Fit at 7–8 mm before declining. TLRE is system-dependent, with side transmission differing from cross-sectional TLRE, which decreases apically without central peaks [38,54]. Longitudinal fiber alignment improves axial conduction [38], while lateral conduction limits side TLRE. In other words, the 1 mm diameter of the side hole opening measured in the current study restricted TLRE measurements to a specific area that faces the radiometer sensor through the post side rather than covering the entire 360-degree circumference of the post.

Radiant exposure depends on light intensity, time, and emission area, with minor losses assumed [38]. Light losses may occur from poor post fit or silicone used. LCU performance is influenced by irradiance, beam uniformity [55,56,57], spectral profile [58,59,60,61,62,63], and ISO standards (≥300 mW/cm^2^, 400–515 nm) [64]. Optimal curing needs 6 J/cm^2^ at 100 mW/cm^2^ [48], while <233 mW/cm^2^ may be considered insufficient [64]. Photoinitiators activate between 380 and 500 nm [60,61,65,66], and tip diameter affects irradiance whereas wavelength remains unchanged [67,68]. Radiometer mismatches or optical variations may explain reduced transmission at deeper levels [69]. In the current study, variations in light transmission may have different explanations. One explanation is that the wavelength sensitivity range of the radiometer must match the wavelength of the curing light used, which may lead to misinterpretation of the amount of light energy that reaches the post system, especially if the post material absorbs or scatters light differently across wavelengths. Another possible explanation is that the radiometers vary in their light collection optics, such as lens curvature, filters, and internal reflectors. These differences can influence how well the device collects scattered, refracted, or low-intensity light, which becomes more relevant at deeper post lengths where TRLE naturally attenuates. Some radiometers may fail to detect the higher or weak light signals accurately at the apical end, giving the impression of a sharper TRLE drop than what occurs.

Glass fiber posts use light- or dual-cure cements, with dual-cure preferred for reliable curing in low-light conditions [26]. Yet light is still required, and low exposure reduces DC [27]. DC rises with light intensity and exposure but also increases stress [40,41,42,43,44]. While 21–24 J/cm^2^ is the optimal radiant exposure for composites [64], thin cement layers may need different values [39]. Conventional and bulk-fill resin composites usually have thicknesses that range from 2 to 6 mm [70,71,72,73,74,75]. In contrast, dental cement applied around prefabricated glass fiber posts is much thinner, typically just a fraction of a millimeter. This variation in thickness means that the radiant exposure required for effective polymerization of dental cement differs from that necessary for thicker resin restorations. Nonetheless, the specific radiant exposure needed to reach optimal DC in dental cement has not yet been established [51]. Polymerization also depends on TLRE [38,39] cement type and spectral match [76,77,78]. Thus, balancing these factors is essential for ensuring the longevity and clinical success of composite resin restorations.

Heat during light-curing is affected by LCU power, irradiance, curing time, and resin properties [65,70,71,72,73,79,80]. Longer curing mainly increases pulpal temperature, while higher irradiance raises surface heat [70]. A rise above 5.5 °C can cause irreversible pulpal damage [74], and 10 °C or more may injure periodontal tissues [75]. High-output LCUs produce greater heat [69,70,81], and during post cementation, temperatures can reach 40–45 °C inside the root canal. This may damage tissues, degrade the post’s optical properties, reduce light transmission, and compromise polymerization. However, more research on heat-related fiber post degradation is needed.

The study has several limitations including restricted range of post systems, reliance on a single LCU, and omission of material and thermal analyses on light transmission. Further studies should simulate clinical conditions, investigate diverse post designs and compositions, and clarify the influence of LCU-induced heat on optical performance.

## 5. Conclusions

Within the limitations of this study, the results demonstrate that the type, configuration, and length of the post system significantly influence TLRE, which consistently decreases with increasing length regardless of the post system used; however, the rate of attenuation varies depending on post design and material. Serrated posts, particularly the EZ-Fit Fiber Post, showed the highest coronal TLRE but the steepest decline with depth. Furthermore, serration may reduce deep light transmission due to fiber discontinuity, which can disrupt light propagation along the post. In contrast, the iLumi Fiber Post exhibited more uniform and stable transmission across lengths, making it more effective in deeper regions. Among smooth posts, RelyX Fiber Post outperformed Easy Post by maintaining higher TLRE values within moderate attenuation, while Easy Post showed consistently low transmission values. These findings further underscore the importance of selecting an appropriate post system based on its material characteristics, light transmission ability, and clinical depth requirements to ensure effective light transmission, optimal polymerization, and improved clinical outcomes in restorative dentistry. Optimizing clinical outcomes therefore requires a thoughtful selection of post systems tailored to the specific needs of each case and the restorative materials being used.

## Figures and Tables

**Table 1:** Characteristics of fiber post systems investigated.

Post System	Shape	Size	Total Length (L)	Coronal Section (L1)	Apical Section (L2)	Coronal Diameter (D1)	Apical Diameter (D2)	Serrated/Wider Section (S1)	Serrated Constructed Section (S2)	Serration Step (SS)	Chemical Composition
Glass Rod	Control, parallel, smooth-sided	Size 1	20 mm	—	—	1.5 mm	1.5 mm	—	—	—	Glass
Easy Post	Tapered, smooth-sided	Size 1	20 mm	13.77 mm	6.23 mm	1.35 mm	0.8 mm	—	—	—	Zirconium-enriched glass fibers 60% and epoxy resin matrix 40%
RelyX Fiber Post	Tapered, smooth-sided	Size 1	20 mm	11 mm	9 mm	1.30 mm	0.8 mm	—	—	—	Zirconium-enriched glass fibers 60% and epoxy resin matrix 40%
EZ-Fit translucent post	Tapered, serrated	Size 2	16.49 mm	5.51 mm	10.97 mm	1.39 mm	0.85 mm	1 mm × 16	0.1 mm	0.1 mm	Glass fibers 77% and epoxy resin matrix 23%
iLumi super fiber post	Tapered, serrated	Size 1	20 mm	14.3 mm	5.7 mm	1.35 mm	0.8 mm	2.3 mm × 4	1.7 mm × 3	0.5 mm	Glass optical fibers 70% and e poxy resin matrix 30%

**Table 2:** The mean radiant exposure transmitted through fiber posts over a 40 s period, along with the peak radiant exposure recorded at the coronal end for each post type and dimension, expressed in mJ/cm^2^.

Post System	Control Glass Rod			Easy Post			RelyX Fiber Post			EZ-Fit Fiber Post			iLumi Fiber Post		
Post size (mm)	1.50 mm			1.35 mm			1.30 mm			1.39 mm			1.35 mm		
Peak intensity at the coronal end (mW/cm^2^)	193.53			193.70			191.80			187.57			170.33		
Dose at the coronal end (mJ/cm^2^)	7824.06			7750.85			7672			7503.14			6813.42		
Post Length (mm)	Mean	SD	Mean (TLRE) mJ/cm^2^(%)	Mean	SD	Mean (TLRE) mJ/cm^2^(%)	Mean	SD	Mean (TLRE) mJ/cm^2^(%)	Mean	SD	Mean (TLRE) mJ/cm^2^(%)	Mean	SD	Mean (TLRE) mJ/cm^2^(%)
7	253.80	11.93	3.01	218.78	22.24	2.82	342.08	21.51	4.45	538.28	7.89	7.17	312.58	5.13	4.58
8	248.07	8.22	3.17	262.29	28.20	3.38	315.87	38.37	4.11	539.85	11.46	7.19	317.99	4.67	4.66
9	235.87	6.86	3.01	198.52	30.94	2.56	385.79	39.45	5.02	489.56	12.59	6.52	331.15	9.24	4.86
10	235.12	11.69	3.01	242.98	18.14	3.31	409.35	20.11	5.33	425.69	7.55	5.67	354.92	15.87	5.21
11	230.77	12.25	2.94	245.31	23.92	3.13	364.04	17.52	4.74	375.82	13.89	5.01	367.97	17.13	5.40
12	228.65	10.33	2.92	231.62	20.60	2.98	376.56	28.87	4.90	391.81	12.73	5.22	338.05	10.97	4.96
13	223.77	7.69	2.86	213.06	24.31	2.74	354.28	24.52	4.61	388.00	3.93	5.17	331.25	8.15	4.86
14	213.37	9.65	2.72	225.36	15.80	2.90	355.66	15.99	4.63	315.92	4.09	4.21	328.92	6.25	4.82
15	207.33	5.01	2.64	197.35	21.63	2.54	312.58	24.88	4.07	276.59	8.42	3.68	304.09	4.42	4.46
16	211.68	9.33	2.70	171.36	12.08	2.21	310.46	22.49	4.04	205.88	10.03	2.74	301.86	6.15	4.43
17	267.17	15.91	3.41	145.68	13.17	1.87	286.90	16.50	3.73	.	.	.	260.80	11.47	3.82
18	237.88	16.00	3.04	146.21	11.63	1.88	235.23	10.07	3.30	.	.	.	242.66	14.06	3.56
19	219.74	10.99	2.80	117.56	19.47	1.51	150.98	22.88	1.96	.	.	.	226.00	7.25	3.31
20	227.17	20.52	2.90	93.37	19.01	1.20	163.18	12.04	2.12	.	.	.	190.24	7.88	2.79

**Table 3:** Model summary for smooth and serrated post systems.

Post System	Model	R	R^2^	Adjusted R^2^	Change Statistics				
					ΔR^2^	F Change	df1	df2	Sig. F Change
Smooth post system	1	0.437 ^a^	0.191	0.189	0.191	98.929	1	418	<0.001
	2	0.651 ^b^	0.424	0.422	0.233	168.768	1	417	<0.001
Serrated post system	1	0.478 ^a^	0.228	0.226	0.228	111.667	1	378	<0.001
	2	0.714 ^b^	0.510	0.507	0.282	216.955	1	377	<0.001

^a^ Predictors: (Constant), post system; ^b^ predictors: (constant), post system, Length from the Coronal End (mm).

## Data Availability

The original contributions presented in this study are included in the article. Further inquiries can be directed to the corresponding author.
